# Effects of Grape Pomace Complete Pellet Feed on Growth Performance, Fatty Acid Composition, and Rumen Fungal Composition in Beef Cattle

**DOI:** 10.3390/ani15070930

**Published:** 2025-03-24

**Authors:** Meimei Teng, Yuanqiu Li, Jiangjiao Qi, Wenda Wu, Xinchang Sun, Chengze Gao, Xia Zhang, Tursunay Mamtimin, Jiangchun Wan

**Affiliations:** 1 Xinjiang Key Laboratory of Grassland Resources and Ecology, College of Grassland Science, Urumqi 830052, China; tengmeimei@outlook.com (M.T.); liyuanqiunord@163.com (Y.L.); 15109936697@163.com (J.Q.); wuwenda@hfut.edu.cn (W.W.); sunxinchang2109@outlook.com (X.S.); gao2645024183@outlook.com (C.G.); zhangxia7938@outlook.com (X.Z.); 2 School of Food and Biological Engineering, Engineering Research Center of Bio-Process, Hefei University of Technology, Hefei 230009, China; 3 Department of Chemistry, Faculty of Science, University of Hradec Kralove, 500 03 Hradec Kralove, Czech Republic; 4 Postdoctoral Station of Grassland Science, Urumqi 830052, China

**Keywords:** non-conventional feed resources, beef cattle, feed efficiency, fatty acid traits, rumen digestive characteristics

## Abstract

Grape pomace, a winemaking byproduct, is nutrient- and polyphenol-rich, but research on its use in beef cattle is limited. This study explored the impact of grape pomace-based complete pellet feed on growth, serum biochemistry, fatty acid profile, and rumen microbiota in beef cattle. Fifteen healthy Simmental cattle were randomly divided into three groups (G0, G15, and G20) and fed a complete pelleted ration containing 0%, 15%, and 20% of grape pomace, respectively, for 60 days. The results showed that the addition of grape pomace to the ration markedly increased the average daily feed intake and average daily weight gain in beef cattle. In terms of biochemistry, the levels of total protein (TP) and albumin (ALB) in the G20 group were higher than in the G0 group (*p* > 0.05). The levels of oleic acid, linoleic acid, and behenic acid were higher in the G20 group than in the G0 group. Grape pomace had no significant effect on rumen fungal diversity and total volatile fatty acids (TVFAs) in beef cattle. The pH and ammonia nitrogen content in the G15 and G20 groups were significantly higher than that in the G0 group. This indicates that grape pomace can be used as feed raw material for beef cattle.

## Simple Summary

Grape pomace, a winemaking byproduct, is rich in sugars (fructose, glucose, and sucrose), proteins, cellulose, organic acids, and other nutrients. Grape pomace has been shown to be used as a feed material for beef cattle. Since information on the chemical composition and nutritional value of grape pomace complete pellet feed is still lacking, in this study, grape pomace was added to the ration to make grape pomace full-price pellet feed for the beef cattle feeding test. The results showed that grape pomace improved the palatability of the ration, increased the average daily intake of beef cattle, regulated blood biochemical indices, and enhanced the immune system of beef cattle. Grape pomace is rich in various bioactive substances, which maintain the balance of rumen microorganisms in beef cattle, increase their fatty acid content, and reduce the cost of rations, thereby improving the economic benefits of farming. This experiment aimed to verify the feeding effect of grape pomace full-value pellet feed on beef cattle breeding to provide a theoretical basis for adding grape pomace to feed.

## 1. Introduction

According to data from the National Bureau of Statistics of China database (NBS, 2022 and 2023), China’s beef production in 2022 and 2023 was 7.1826 million tons and 7.5268 million tons, respectively. However, China’s beef imports reached 2.6899 million tons and 2.7374 million tons, respectively, during the same period, representing a 1.77% increase compared to the previous year’s imports, indicating that domestic production is unable to meet the growing demand for beef. At present, China’s beef industry currently faces multiple technical challenges, including the shift from traditional feeding methods, feed resource scarcity, and escalating raw material costs. All of them adversely affect the development of livestock [1]. Therefore, developing non-conventional feed resources is crucial for mitigating feed shortages, cutting costs, and sustaining livestock growth. With this background, studies have found that fruit and vegetable residues, such as apple pomace, pomegranate pomace, sugarcane tops, banana rhizome bulbs, citrus by-products, tomato pomace, grape pomace, carrot pomace, and potato waste, have been found to be suitable as feed ingredients for ruminants [2,3].

Notably, Xinjiang, China’s grape-growing hub, produces vast amounts of grape pomace from winemaking. Grape pomace, made of seeds, skins, and stalks, can be used as a raw material for animal feed to boost digestive function [4,5]. It is also rich in polyphenols such as tannins, proanthocyanidins, anthocyanidins, flavonoids, and phenolic acids, which have the potential to enhance the antioxidant capacity [6,7]. The antioxidant properties of grape pomace phenolics are mainly derived from their free radical scavenging ability, the role of phenolic hydroxyl groups, and structural properties. Juráček et al. (2021) [8] found that adding 1% and 2% grape pomace to the diet of rams reduced blood glucose concentrations with no impact on other blood biochemical indicators. Previous studies have verified that polyphenols can be added to ruminant diets [9,10]. The study by Molosse et al. (2023) [11] showed that grape pomace did not affect the initial body weight (IBW) and final body weight (FBW) of beef cattle but was beneficial to health, modulating antioxidants, and the immune system. During muscle storage, reactive oxygen species (ROS) are generated, and these reactive oxygen species tend to cause the oxidation of lipids [12]. Therefore, feeding dietary materials with antioxidant properties is an important way to enhance the oxidative stability of muscles [13].

Complete pellet feed, formulated by proportionally mixing concentrates and roughage, provides comprehensive nutrition, palatability, mildew resistance, and ease of storage and transport while improving feed grain utilization [14]. The rumen microbial community consists mainly of bacteria, fungi, and protozoa, with the proportion of fungi being significantly influenced by the cellulose content in the diet. When the cellulose content in the diet is higher than that of starch and grains, the population of rumen fungi increases accordingly [15,16]. However, current studies on grape pomace primarily focus on its use in sheep production and its influence on milk production in dairy cattle, and most of the studies on rumen flora have focused mainly on bacteria, but this study attempted to analyze the effect of grape pomace-derived cellulose and lignin on rumen microorganisms in terms of fungal communities [17,18]. In view of the shortage of forage raw materials and the inconvenience of feeding during winter in Xinjiang [19], this study aims to determine the optimal grape pomace inclusion in complete pellet feed. It evaluates the effects on growth performance, blood biochemical indices, muscle fatty acid content, and rumen microorganisms in beef cattle, providing a theoretical basis for grape pomace utilization in feed.

## 2. Materials and Methods

### 2.1. Materials and Determination of the Nutritional Level of the Ration

The Cabernet Sauvignon grape pomace used in this research, sourced from Xinjiang CITIC Guoan Wine Industry Company Limited’s Manas County Branch (Changji, China), is sourced from the natural drying of grape pomace to not less than 10% moisture content and stored in a dry, cool place. Its nutrient composition is outlined in Table 1. The grape pomace material and the diets in Table 2 were dried in an oven at 65 °C for 48 h until constant weight, crushed, and sieved (0.425 mm). Their dry matter (DM), crude protein, fat, and ash contents were then analyzed using the Association of Official Agricultural Chemists method [20]. Their neutral and acid-detergent fiber contents were made according to the method proposed by Vansoest et al. (1991) [21]. Condensed tannin was determined using the Amarowicz et al. (2004) [22] method.

### 2.2. Animal Management and Experiment Design

This experiment was conducted at Xinjiang Jinguancheng Biotechnology’s farm from December 2023 to February 2024 (Xinjiang, China). Fifteen 13-month-old healthy Simmental crossbred beef cattle with an average weight of (average weight: 287.39 ± 3.75 kg) were randomly divided into three groups of five, with 7 days for the adaptation period and 60 days for the experimental period. The control group (Group G0) and the experimental groups (G15 and G20 Groups) were fed complete pellet feed containing 0%, 15%, and 20% grape pomace, respectively. The nutrient composition of this complete pellet feed was formulated according to the “China Feeding Standard of Beef Cattle” (NY/T 815-2004 [23]) to meet the growth requirements of animals. All the feed Materials were crushed in a pulverizer (HL 650-1300, Zouping Hanlong Machinery Manufacturing Co., Ltd., Binzhou, China), and, according to the ratios of diet composition listed in Table 2, the roughage and concentrate were mixed and directly processed into complete pelleted feed with a particle size of 4.6 mm using a feed pelletizer (9KL, manufactured by Qufu Shengtai Machinery Co., Ltd., Qufu, China).

During the experiment, test animals were dewormed, housed in a clean barn, fed at 09:30 and 17:30, and had ad libitum water access.

All the experimental procedures were approved by the Ethics Committee at the Experimental Animal Welfare of Xinjiang Agricultural University (approval number. 20221489).

### 2.3. Determination of Growth Performance

At the beginning and end of the trial, the test beef cattle were weighed after 12 h of fasting and water deprivation, and the following formula was used to obtain the average daily gain (ADG) based on their IBW and FBW:

ADG = (FBW − IBW)/number of test days

During the experiment, the total feed intake and residual test beef cattle cow in each treatment group were recorded to calculate the average daily feed intake (ADFI). Based on the ADG and the average daily feed intake data, the following formula was used to determine the feed conversion ratio (F/G):

ADFI (kg/day) = [total feed given during the test period in each group (kg) − total leftovers (kg)]/number of test days (day);

F/G = DMI/ADG

### 2.4. Blood Biochemical Analysis

Before weighing beef cattle on day 60, three beef cattle were randomly selected from each group, and blood was collected through the tail vein using ethylene diamine tetraacetic acid blood collection tubes. The samples were then centrifuged at 4752× *g* for 15 min to prepare serum samples, which were stored at −60 °C for reserve. The level of urea nitrogen (UN), total protein (TP), albumin (ALB), triglycerides (TGs), and total cholesterol (TCHO) were evaluated using a fully automated biochemical analyzer (Myriad BS-420, Shenzhen Mindray Bio-Medical Electronics Co., Ltd., Shenzhen, China).

### 2.5. Fatty Acid Content in Muscle

Post-blood collection, three beef cattle in each group were randomly selected for slaughter, and the beef samples were taken from the meat samples of the longest dorsal muscle between the 10th and 12th ribs on the left side [24]. The sample (0.05 g) was placed on ice, thawed, and ground. After adding 150 μL of methanol, 200 μL of methyl tertiary-butyl ether, and 50 μL of 36% phosphoric acid, the mixture was vortexed for 3 min and centrifuged at 4 °C for 5 min at 12,000 rpm. For the extraction method, refer to Dannenberger et al. (2013) [25]. Briefly, the supernatant was dried using a nitrogen blower, and then 300 μL of 15% boron trifluoride methanol solution was added. The sample was vortexed and incubated at 60 °C for 30 min. After cooling, 200 μL of saturated sodium chloride and 500 μL of n-hexane were added, followed by centrifugation and vortexing. Finally, 100 μL of the n-hexane layer was pipetted for gas chromatography (GC) detection (7890B-7000D, Agilent, Santa Clara, CA, USA). The analysis was conducted using a gas chromatograph equipped with a chromatographic column (30 m × 0.25 mm × 0.25 μm, DB-5MS, 7890B-7000D, Agilent, Santa Clara, CA, USA), with a non-split injection volume of 1 μL and a column flow rate of 1 mL/min. Oven Temperature Ramp: 40 °C held for 2 min, raised to 200 °C at a rate of 30 °C/min and held for 1 min, raised to 240 °C at a rate of 10 °C/min and held for 1 min, raised to 285 °C at a rate of 5 °C/min and held for 3 min.

### 2.6. Determination of Rumen Fermentation Parameters

The test cattle were fasted for 12 h. Three beef cattle were randomly selected from each group, and after slaughter, the rumen fluid was collected, and then the collected rumen fluid was filtered with six layers of sterilized gauze. The pH of the rumen was determined using an acidimeter (Model Z-201F; Shanghai Yidian Scientific Instrument Co., Ltd., Shanghai, China). The colorimetric method using phenol-hypochlorite estimates the amount of ammoniacal nitrogen (NH_3_-N) [26]. Rumen protein (microbial protein; MCP) content was determined using the Caulmers Brilliant Blue method, and volatile fatty acid (VFA) content was determined using gas chromatography (GC-2010; Shimadzu Enterprises Management Co., Ltd., Urumqi, China) [27]. Chromatographic conditions: using a DB-FFAP flexible quartz capillary column (30 m × 0.25 mm × 0.25 μm) and a split injection volume of 5 μL, the initial column heater temperature was 50 °C for 1 min. Subsequently, the temperature was heated up to 120 °C at a rate of 15 °C/min and then to 240 °C at 5 °C/min and left on for 3 min, maintaining the inlet temperature at 250 °C. Finally, Nitrogen was used as the carrier gas at a flow rate of 1.0 mL/min, and the splitting ratio was 50:1.

### 2.7. Rumen Microbial Community Analysis

This fluid was filtered through six layers of sterilized and disinfected gauze and centrifuged for 3 min (9000× *g*; 4 °C) after adding phosphate-buffered saline. Rumen microbial DNA was extracted according to the manufacturer’s instructions for the MP Fecal DNA Extraction Kit (MP Biomedicals, Shanghai, China). DNA purity was measured using a NanoDrop2000 (Thermo Scientific, Waltham, MA, USA). The DNA concentration was measured using TBS-380, and DNA was broken into fragments of about 200~500 bp using an ultrasonic crusher (Covaris M220, Gene Company Limited, Beijing, China). Fungi with a length of about 251 bp were selected. The ITS V1 region was used as the measurement region. The primers ITS5F (5′-GGAAGTAAAAGTCGTAACAAGG-3′) and ITS2R (5′- GCTGCGTTCTTCATCGATGC-3′) were used for the PCR amplification process (2720, Applied Biosystems, Carlsbad, CA, USA). PCR products were detected using 2% agarose gel electrophoresis, purified using the Quant-iT PicoGreen dsDNA Assay Kit (Invitrogen, Thermo Fisher Scientific, Dallas, OR, USA), and read on a Microplate reader (FLx800, BioTek, Seattle, WA, USA). Using Quant-iT PicoGreen dsDNA Assay Kit, PCR products were quantified using a Quant-iT PicoGreen dsDNA Assay Kit on a microplate reader (FLx800, BioTek, Seattle, WA, USA). Library construction was then performed using the TruSeq Nano DNA LT Library Prep Kit (Illumina, San Diego, CA, USA). High-throughput sequencing was performed on an Illumina NovaSeq machine (Illumina, Carlsbad, CA, USA) using the NovaSeq 6000 SP Reagent Kit (500 cycles). The rumen fluid samples were preserved in dry ice and sent to Shanghai Paixin Genetic Science and Technology Co. (Shanghai, China) for sequencing. Data processing was performed on the Parnassian Cloud platform, and the original data were submitted to NCBI, accession. no: PRJNA1192575.

### 2.8. Statistical Analysis of Data

The SPSS software (version 28.0) was utilized to conduct a one-way ANOVA with a comparison of means using Tukey’s test. The data were presented as the mean ± standard error, and statistical significance was set at *p* < 0.05. Initial weight was modeled as a covariate, but it resulted in a non-significant value (*p* > 0.05) and was subsequently excluded. The beta diversity of rumen fungi was analyzed using permutation tests for statistical significance. Alpha diversity and relative abundance were examined using a one-way ANOVA. Heatmaps were created in Origin software (version 21.0) using correlation plots (*p* < 0.05), and correlation analysis was evaluated based on Spearman’s correlation coefficient.

## 3. Results

### 3.1. Growth Performance

Table 3 shows the growth performance of beef cattle. There were no differences (*p* > 0.05) in IBW, FBW, and F/G ratio among all groups in terms of feed intake and growth performance. The DMI was higher (*p* < 0.05) in the G20 group compared to the G0 group. The G20 group demonstrated an increased (*p* < 0.05) average daily weight gain in comparison to the G0 and G15 groups.

### 3.2. Blood Biochemical Analysis

Table 4 shows the results of the blood biochemical analysis of the cattle. The levels of TP and ALB were higher (*p* < 0.05) in the G20 group than in the G0 group. The proportion of GLB, ALB/GLB, ALB/TP, GLB/TP, TGs, and TCHO was not significantly different among beef cattle groups (*p* < 0.05).

### 3.3. Fatty Acid Composition of Beef Cattle Muscle

The evaluation of the fatty acid composition (Table 5) showed that levels of saturated fatty acids (SFAs) and Octanoic acid (C8-0) in beef cattle muscle were increased (*p* < 0.05) in the G15 group compared with the other groups. The concentrations of arachidic acid (C20-0) in the G15 and G20 groups were higher (*p* < 0.05) compared to the control group. Levels of palmitic (C16-0), stearic (C18-0), and saturated fatty acids were higher (*p* < 0.05) in the 1G15 and G20 groups compared to the G0 group. Among monounsaturated fatty acids (MUFAs), the levels of myristoleic acid (C14-1) and oleic acid (C18-1n9c) in the G20 group were higher (*p* < 0.05) than in the G0 group. MUFA content in the G20 group was higher (*p* < 0.05) than that in the G0 and G15 groups by 34.94% and 21.80%. Among the polyunsaturated fatty acids (PUFAs), γ-linolenic acid (C18-3n6) levels in the G15 and G20 groups were higher (*p* < 0.05) than that in the G0 group. The levels of linoleic acid (C18-2n6c) and PUFAs were higher (*p* < 0.05) in the G15 and G20 groups compared with those in the G0 group.

### 3.4. Rumen Fermentation Parameters in Beef Cattle

**Figure 1:**
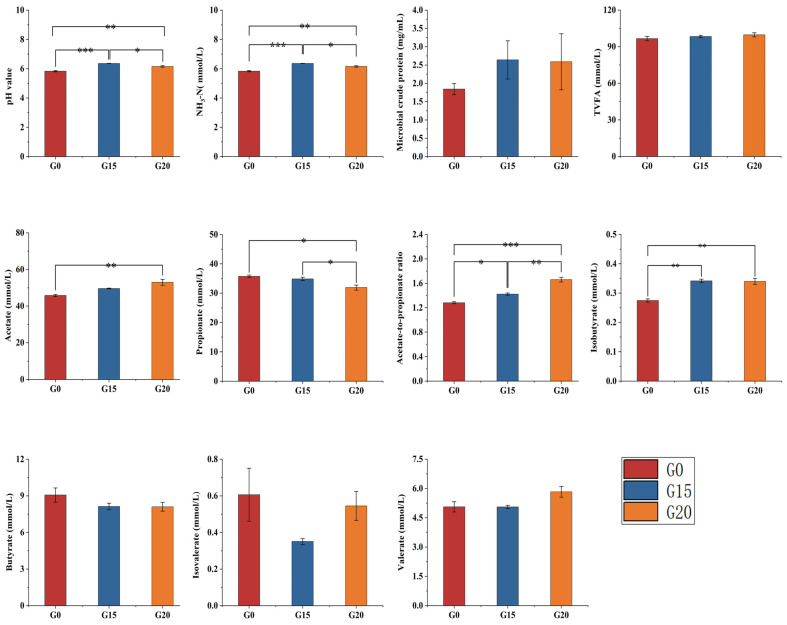
Effects of grape pomace complete pellet feed on rumen fermentation parameters of beef cattle. G0: the control group, which consists of the complete pellet feed containing 0% grape pomace; G15: the 15% grape pomace complete pellet feed; G20: the 20% grape pomace complete pellet feed. Data analyzed by Tukey, presented as mean ± SEM, with * for *p* < 0.05, ** for *p* < 0.01, *** for *p* < 0.001 significance.

Figure 1 shows the values of rumen fermentation parameters in beef cattle. Compared to the G0 group, the pH and NH_3_-N were increased (*p* < 0.05) in the G15 and G20 groups. Acetic acid content and acetic/propionic acid ratio levels in the G20 group were higher (*p* < 0.05) than in the other groups. Additionally, propionic acid levels in the G20 group were lower (*p* < 0.05) than in the other groups. The levels of isobutyric acid in the G15 and G20 groups were higher (*p* < 0.05) than those in the control group. No differences (*p* > 0.05) were seen in the other rumen fermentation parameters.

### 3.5. Effects of the Rumen Microbial Community

**Figure 2:**
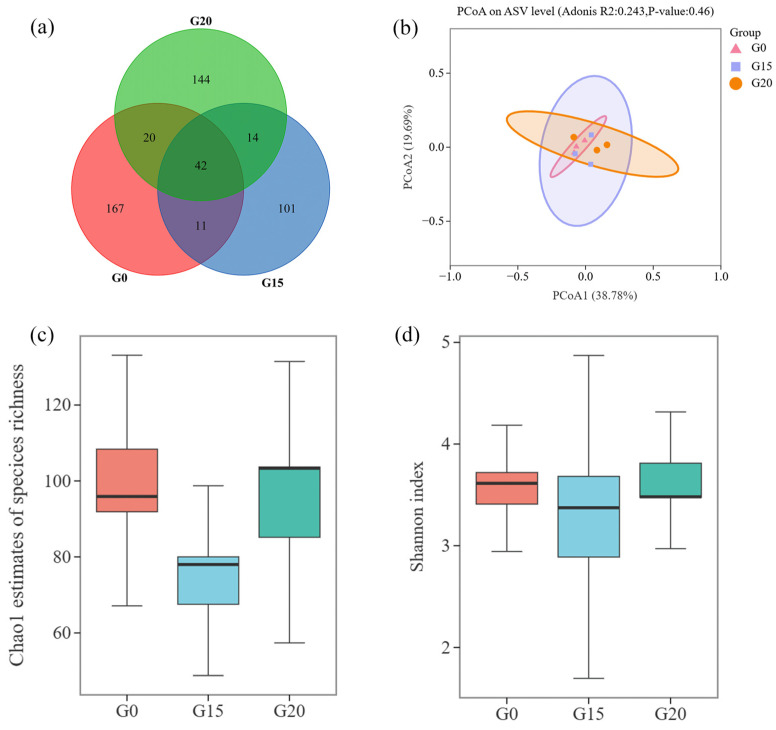
Effects of different levels of grape pomace complete pellet feed on ASVs, and beta and alpha diversity of rumen fungi in beef cattle. (**a**) ASVs; (**b**) Principal component analysis; (**c**,**d**) Richness (Chao1 index) and diversity (Shannon index) within the rumen microbial communities of different treatment groups.

After filtering out low-quality and denoised sequences, 1,170,719 valid sequences remained, averaging 130,080 entries per sample and a sequence length of 252 bp. Across the three groups, 42 amplicon sequencing variants (ASVs) were shared, with 240, 168, and 220 ASVs in the G0, G15, and G20 groups, respectively. The G0 group had the most ASVs, while the G15 group had the least (Figure 2a). The principal coordinate analysis (PCoA) using the Bray–Curtis distance at the ASV level for rumen fungal groups is depicted in Figure 2b. Principal Components 1 and 2 contributed 38.78% and 19.69%, respectively. The overlapping projections indicate that there were no differences (*p* > 0.05) in beta diversity among the G0, G15, and G20 groups. As shown in Figure 2c,d and Table S1, the inclusion of grape pomace did not affect (*p* > 0.05) the Chao1 or Shannon indices.

**Figure 3:**
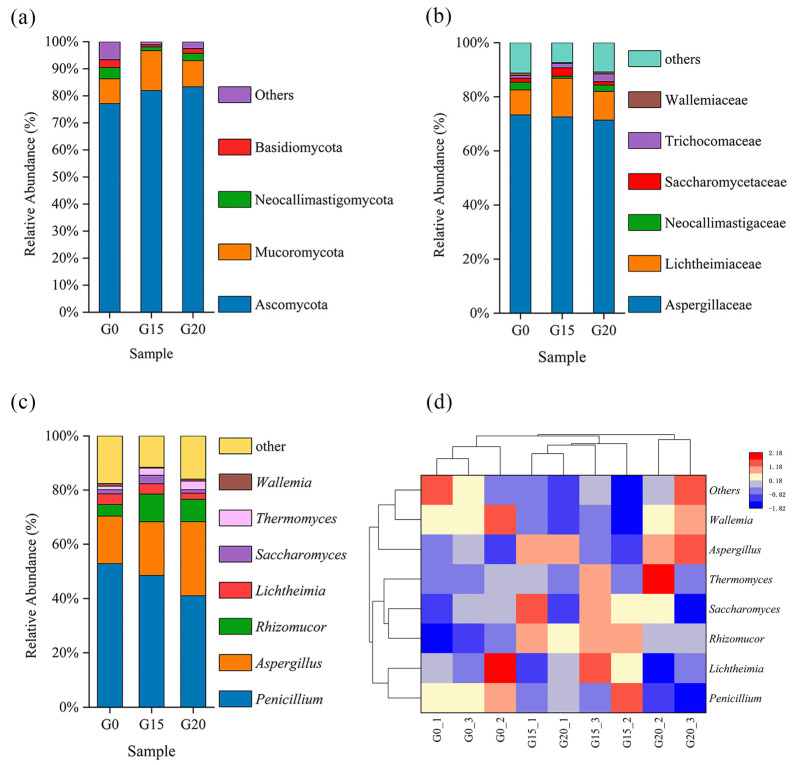
Effect of grape pomace complete pellet feed on rumen microbial composition. Community differences at the phylum (**a**), family (**b**), and genus (**c**,**d**) levels. G0: control group; G15: 15% grape pomace-treated group; G20: 20% grape pomace-treated group.

At the taxonomic level, the samples contained 7 phyla, 16 classes, 28 orders, 42 families, 68 genera, and 111 species. Ascomycota, Mucoromycota, and Neocallimastigomycota were the dominant phyla, comprising 90.43% (G0 group), 98.09%, and 95.71% (G15 and G20 groups) of the total rumen fungi (Figure 3a; Table S2). Compared to the G0 group, the relative abundance of the Ascomycota phylum increased (*p* < 0.05) in the G20 and G15 groups. The relative abundance of the (Mucoromycota) phylum was higher (*p* < 0.05) in the G15 than that in the G0 and G20 groups, while Neocallimastigomycota showed no difference (*p* > 0.05) among the three groups.

At the family level, Aspergillaceae, Lichtheimiaceae, and Neocallimastigaceae were dominant (Figure 3b; Table S3). The relative abundance of Lichtheimiaceae in the G15 group was higher (*p* < 0.05) than that in the G0 and G20 groups.

At the genus level, the 20% grape pomace complete pellet feed group had a higher (*p* < 0.05) relative abundance of *Aspergillus* than the control diets and 15% groups (Figure 3c,d; Table S4). The G0 group had a higher (*p* < 0.05) relative abundance of *Rhizomucor* than the G20 and G15 groups, while other flora showed no effects (*p* > 0.05).

**Figure 4:**
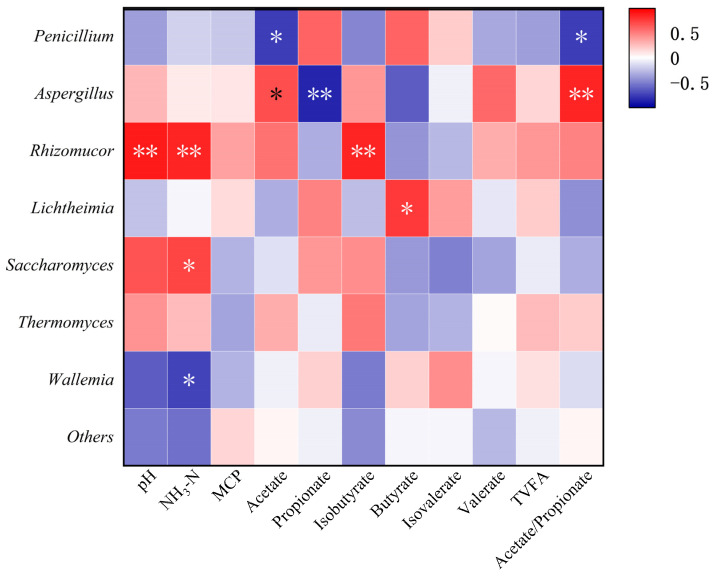
Correlation between rumen microorganisms and fermentation parameters in beef cattle. Note: * and ** denote statistical significance at the *p* < 0.05 and *p* < 0.01 levels of correlation, respectively.

The correlation between the relative abundance of fungal genera and rumen fermentation parameters in beef cattle is shown in Figure 4. Rhizomucor had a positive correlation with the pH, NH_3_-N, and isobutyric acid levels. The acetic acid content and acetic/propionic acid ratio exhibited a significant negative correlation with the *Penicillium*. There was a notable positive correlation between the *Aspergillus* and both the level of acetic acid and the ratio of acetic acid to propionic acid. Propionic acid content showed a significant negative correlation with the *Aspergillus*. In addition, *Lichtheimia* was positively associated with butyrate, while *Saccharomyces* abundance showed a significant positive correlation with NH_3_-N levels. In contrast, the relative abundance of *Wallemia* was significantly and negatively correlated with NH_3_-N levels.

### 3.6. Economic Benefits

As shown in Table 6, the unit prices of feed (DM basis) for the G0, G15, and G20 groups were 2.60, 2.09, and 2.04 CNY/kg. The G20 group had the highest total feed intake (562.51 kg/head). The feed costs for G15 and G20 were 1112.24 and 1147.52 CNY/head, respectively, 15.17% and 11.61% lower than G0. Weight gain income was 1106.62, 1245.85, and 1663.73 CNY/head for G0, G15, and G20, respectively, with the highest in G20. G15 and G20 profits were 133.61 and 516.21 CNY/head, while G0 had a loss due to market price fluctuations.

## 4. Discussion

Grape pomace contains various bioactive compounds and nutrients that can improve animal health, growth, and development [28]. The wine-fermented grape pomace was pelletized at high temperatures and had an aroma that made the ration palatable, thus increasing its average daily feed intake, which is consistent with Tayengwa et al.’s. (2020) [29] findings. In this study, grape pomace addition had no effect on feed conversion due to the same nutrient composition among the three groups of rations, which is consistent with the results of Molosse et al. (2023) [11]. Caetano et al. [30] found that in Angus bulls, they reduced the FBW and ADG, despite the DMI and F/G in the grape pomace group being higher than those in the control group. This disagrees with our suggestion in the report, possibly due to the previous study in which grape pomace was added at a rate of 300 g/kg. The nutrient content of the feed in the experimental group was higher than that of the control group; the highest rate in the present study was 20%, and the nutrient content was the same in all groups, but there were inter-individual differences, which influenced led to variability in the results. The above study showed that grape pomace could be incorporated into ruminant raw material.

The liver serves as the primary site for blood biochemical indicators that indirectly reflect animal growth and development, as well as nutrient absorption and utilization [31]. TP in serum consists of ALB and GLO, which reflects the animal’s protein synthesis capacity and metabolic level. It also performs crucial roles in maintaining blood osmotic pressure, pH balance, metabolite transportation, and immune system regulation [32]. In contrast, grape pomace had no significant effect on the serum levels of α-lactalbumin, caseins, and ALB in dairy cows [33]. This study is inconsistent with the results of the appeal study and may be linked to factors such as protein intake, energy demands, and liver functionality [34]. This study concurs with previous research in lamb that grape pomace silage increased serum TP levels [35]. This research shows that grape pomace can improve blood lipid levels and enhance the overall health of animals. The liver of animals primarily synthesizes TCHO and TG, which are indicators of fat metabolism [36]. In our study, various proportions of grape pomace in complete pellet feed for beef cattle did not significantly alter TCHO and TG levels, suggesting no adverse impact on their fat metabolism. Alba et al. (2019) have reported that grape pomace increased serum TG levels in lactating dairy sheep in heat stress, but it had no significant effect on TCHO [37]. Different amounts of grape pomace added, along with variations in animal types and feeding times, result in disparities in lipid and unsaturated fat intake.

The content and composition of fatty acids reflect the nutritional and food value of animal muscle, influencing meat flavor and being crucial for human health [38]. Muscle fatty acids are divided into SFAs and unsaturated fatty acids (UFAs), with SFAs providing ample energy for animals’ daily activities, growth, and development [39]. Bennato et al. (2023) showed that 10% grape pomace decreased palmitic acid levels while tending to increase stearic acid and SFAs in lamb muscles [40]. Our study found that incorporating different amounts of grape pomace into complete diets significantly boosted the palmitic, stearic, and SFAs in beef cattle muscle. The difference between the current study and previous research was due to variations in diet composition and animal species. Previous studies fed lambs a pelleted diet with 10% grape pomace, primarily consisting of grains, soy, oil, vitamins, and minerals. In contrast, our study fed beef cattle diets with 15% and 20% grape pomace, incorporating forage grains such as corn, wheat bran, soy, salt, premix, and others. UFAs are classified into MUFAs and PUFAs. MUFAs can reduce the cholesterol content of the animal and improve the meat flavor, with oleic acid being its primary content [41,42]. We found that the oleic acid and MUFA contents increased with the addition of grape pomace. Arend et al. (2022) discovered no impact on MUFA and oleic acid levels in beef cattle muscle when incorporating 54% grape pomace into their feed, which differed from the results of the present study [43]. This is probably due to the composition of the diets and the amount of grape pomace added, which resulted in diets containing different polyphenolic compounds, flavonoids, resveratrol, and anthocyanins, which in turn affected the different fatty acid types and contents of the muscles [44]. PUFAs contribute to maintaining heart health and reducing the risk of cardiovascular diseases, thereby exerting a significant influence on human diet and health [45]. Molosse et al. (2024) found that different processed grape pomace did not significantly alter the PUFA, linoleic acid, and γ-linolenic acid contents in beef cattle muscle [46]. However, different proportions of grape pomace complete pellet feed fed to beef cattle increased PUFA, linoleic acid, and γ-linolenic acid contents in the muscle in the present study. Adding grape pomace at 8%, 16%, and 24% to lamb diets increased the PUFA, linoleic acid, and γ-linolenic acid contents in the muscle [47]. This enhancement could be attributed to grape pomace’s high phenolic content, which possesses antioxidant properties that minimize PUFA oxidation into volatile compounds [48]. Tannins inhibit rumen microbial activity, reduce rumen biohydrogenation processes, promote fatty acid deposition, inhibit the hydrogenation of C18:2n-6 and C18:3n-3 from polyunsaturated fatty acids to C18:0 from saturated fatty acids, and reduce bacterial growth substrates directly related to rumen fat metabolism, altering rumen fermentation parameters [47,49]. It has been found that tannins have a regulatory effect on rumen fermentation and affect the content of polyunsaturated fats in muscle [50]. Xiong et al. (2021) [51] found that paper mulberry silage contains tannins, and the addition of paper mulberry reduced the abundance of Helicobacter phylum in the test group, thus affecting muscle fatty acid content. Polyunsaturated fatty acid content was found to be increased with the paper. The polyunsaturated fatty acid content increased with increased paper mulberry addition. Hennessy et al. (2021) [52] found that the addition of n-3 polyunsaturated fatty acid supplementation to the ration of beef cattle had no effect on the monounsaturated fatty acid content of beef muscle and significantly increased the polyunsaturated fat content. Muscle fat content was affected by pre- and post-slaughter, as well as by management factors and the environment [53]. Some studies have found that feeding pasture can increase the polyunsaturated fatty acid content of omega-3, alpha-linolenic acid, and other polyunsaturated fatty acids in the muscle of beef cattle [54,55]. The test cattle used in this experiment were grazed on natural grassland before the start of the experiment, foraged on forage such as needle fescue and duck fescue, and were fed an added grape pomace ration at a later stage; it is possible that the above factors influenced the changes in muscle PUFA values and MUFA content, and the exact reasons for this need to be further explored.

Ruminal fermentation is a process in which feed substances ingested by ruminants are degraded and fermented by rumen microorganisms, affecting the normal growth and metabolism of ruminants [56]. Rumen pH, NH_3_-N, MCP, and volatile fatty acids (VFAs) are key indicators for assessing rumen fermentation status [57]. A rumen pH below 5.6 persisting for 3 h/day induces subacute ruminal acidosis (SARA) and disrupts microbial activity [58,59]. Diets with high-concentrate proportions contain non-structural carbohydrates that cause drastic pH drops in the rumen, triggering acidosis [60,61]. Ren et al. (2024) [62] observed that lamb rumen fluid pH remained above 5.6 in both groups fed two types of complete pelleted diets. Consistent with our findings, complete pelleting does not compromise rumination or predispose animals to SARA despite grinding before pelleting. In this study, the lower pH in group G0 compared to the other two groups might be associated with the concentrate proportion in their diet. The NH_3_-N content in rumen fluid reflects the balance between nitrogenous feed degradation by microorganisms and MCP synthesis [63]. Ruminal MCP concentration is an essential energy source for ruminants as it directly affects feed utilization [64]. In this study, grape pomace was able to significantly increase the content of NH_3_-N, and there was an increasing trend in the content of microbial proteins, but there was no statistically significant difference. Due to the substitution of alfalfa hay and wheat straw by grape pomace in this study, a reduction in rumen energy supply may have resulted. Microbial utilization of peptides, amino acids, and NH_3_-N integration is limited, affecting microbial protein content [65]. Peptides and amino acids in excess of microbial requirements are further catabolized due to insufficient ATP supply, thereby releasing more ammonia nitrogen into the rumen [66]. With the increase of grape pomace addition, the lignin content contained in the ration increased, leading to a decrease in rumen energy supply, thus limiting the microbial uptake and utilization of peptides, amino acids, and ammonia nitrogen from the ration, resulting in an increase of ammoniacal nitrogen content in the rumen. TVFA is the primary energy source for ruminants and primarily comprises acetic, propionic, and butyric acids [67]. Guerra-Rivas et al. (2017) conducted an in vitro digestive assay with sheep and found that, when grape pomace was added, it significantly reduced the levels of TVFA, propionic acid, and butyric acid levels as well as increased the ratio of acetic/propionic acid [68]. In this study, incorporating grape pomace into the diet raised acetic acid levels and the acetic/propionic acid ratio. However, the NH_3_-N declined as the grape pomace proportion increased. TVFA and butyric acid levels remained unaffected. The polyphenols present in grape pomace have an effect on rumen fermentation. Norris et al. (2020) [69] found that the rumen NH_3_-N of bulls showed a decrease with the addition of tannin extract to the ration. Kara et al. [70] found that the addition of grape pomace at 22.5% lowered NH_3_-N.

The rumen, as a unique digestive organ in ruminants, contains a complex and diverse microbial community, including bacteria, protozoa, and fungi [71]. Fungi constitute 5–20% of rumen microorganisms and possess enzymes like cellulases, xylanases, and hydrolases to degrade lignocellulose in feed by secreting these enzymes [72]. Moreover, fungal metabolites support the growth of other microorganisms, enhancing ecosystem stability and functionality [73]. Ascomycota, a key rumen fungus, leads the fungal community in breaking down lignin and keratin [74]. Mucoromycota boosts ruminant digestion and health by balancing microbes and improving feed use [75]. Neocallimastigomycota, featuring robust mycelia and mycorrhizae, converts lignified fibers, proteins, and starch into absorbable nutrients [76]. Wang et al. (2023) found that the rumen of ruminants was dominated by Ascomycetes, Trichoderma, and Neolithic flagellates [77]. Consistent with our study, ration composition likely influenced rumen microbe degradation, affecting beef cattle flora [78]. Grape pomace contains active substances such as tannins, which inhibit the members of the Neolithic flagellate phylum [79]. Khiaosa-Ard et al. (2024) found that grape pomace did not affect alpha diversity but influenced the beta diversity of rumen fungi [80]. In the present study, grape pomace had no effect on beef cattle rumen fungi diversities. *Penicillium* and *Aspergillus* produce enzymes to enhance feed digestion [81]. The results of this study showed that grape pomace had no significant effect on *Penicillium*. It significantly increased the relative abundances of *Aspergillus* and *Rhizomucor*, which may be influenced by the processing of grape pomace. A previous study reported that using different methods, such as yeast and organic fermentation during winemaking, affects the changes in the proportions of *Aspergillus* and *Penicillium* [82].

Rumen fluid pH maintains the normal metabolism of rumen microorganisms, and either a high or low pH affects rumen microbial activity [83]. NH_3_-N provides a nitrogen source for rumen microbes to synthesize MCP [84]. Beef cattle rumen analysis shows a pH and NH_3_-N link to Rhizomucor abundance, possibly due to grape pomace’s bioactives boosting its activity in the complex rumen ecosystem [85]. In this study, Penicillium abundance negatively correlated with acetic acid and acetic/propionic acid ratio, possibly due to nutrient competition with rumen bacteria, and VFAs like acetic and propionic acids are influenced by diet, microbial species, and activity [86,87,88]. High acetic acid ratios indicate VFA bacteria dominance, hindering *Penicillium* growth and abundance. Sosa et al. [89] added Aspergillus to alfalfa grass in vitro and found that it increased acetic acid and acetic/propionic acid ratio in rumen VFAs, with no significant impact on propionic acid. Kong et al. [90] found the study to be consistent with the findings of previous studies. This study found a significant positive correlation between acetic acid levels, acetic/propionic acid ratio, and *Aspergillus* abundance, whereas propionic acid content showed a significant negative correlation. Acetic and propionic acids, short-chain fatty acids derived from amino acid metabolism, may increase due to *Aspergillus’* protein hydrolysis activity, boosting acetic acid levels [91].

Finally, we added grape pomace to the feed ration with a grape pomace raw material price of CNY0.15/Kg, and the G20 group had the highest weight gain income and the highest profit. Molosse et al. [11] found that the addition of grape pomace silage to the feed ration resulted in a reduction of feed prices by 6.25% and an increase in profit by 13.00%. Thus proving that adding grape pomace to beef cattle feed rations is economically viable. The large-scale production of wine has increased the difficulty of managing grape pomace, as traditional disposal methods such as incineration or heat dissipation can easily lead to environmental pollution [92]. However, utilizing grape pomace as a feed ingredient not only avoids impacting wine prices but also provides wine producers with additional economic benefits from the byproduct [93]. There is no fundamental conflict between using grape pomace as cattle feed and wine production. On the contrary, through resource recycling, it achieves a win-win situation for both environmental and economic benefits. The relationship between the two is essentially a collaborative synergy between upstream and downstream sectors of the industry chain rather than competition for resources [94]. Considering that some people are not comfortable with wine or grapes, we may also consider using labels such as “no wine product” or “no wine ingredients” to ensure that consumers are aware that their food does not contain any wine or alcohol.

## 5. Conclusions

In this study, the optimal addition ratio of grape pomace was suggested to be 20%, which indicates that grape pomace does not have adverse effects on the growth performance of beef cattle. The grape pomace can regulate the levels of total protein and albumin in the blood lipids of beef cattle, improve their immunity, as well as increase the linoleic acid and γ-linolenic acid contents in their muscles. Additionally, it increased the pH of the fermentation parameter and elevated the NH_3_-N content without negatively affecting rumen fermentation. Furthermore, grape pomace had no significant effect on the alpha and beta diversities of rumen fungi in beef cattle but significantly increased the relative abundance of rumen Ascomycota and Trichoderma, which was beneficial to the animals’ health. The total cost of feeding rations decreased as grape pomace increased. These results indicate that grape pomace by-products can be effectively used as feed ingredients for beef cattle.

## Figures and Tables

**Table 1:** Nutrients of grape pomace (dry matter basis).

Items	Content
Dry matter/(DM/%)	93.50
Crude protein/(DM/%)	13.11
Crude fat/(DM/%)	12.70
Neutral detergent fiber/(DM/%)	38.10
Acid detergent fiber/(DM/%)	30.82
Ash/(DM/%)	8.50
Condensed tannin/(g/kg)	2.51

**Table 2:** Chemical composition and nutrient levels of the experimental diets (air-dry basis; %).

Items	Groups		
	G0	G15	G20
Corn	42.12	39.04	37.17
Wheat bran	6.79	10.34	12.96
Soybean meal	7.19	4.87	3.88
Salt	1.00	1.00	1.00
Premix ^1^	1.00	1.00	1.00
Corn bran	1.96	1.05	0.65
Alfalfa hay	12.32	3.66	0.00
Corn straw	11.52	7.34	6.12
Grape pomace	0.00	15.00	20.00
Wheat Straw	12.17	7.64	8.00
Corn cob	3.93	9.05	9.22
Total	100.00	100.00	100.00
Chemical composition (g/kg DM)			
Combined net energy (NEmf) ^2^, MJ/kg	6.03	6.02	6.03
Crude protein (CP), % of DM	11.13	11.15	11.16
Crude fat (CF), % of DM	2.6	3.31	3.58
Neutral detergent fiber (NDF), % of DM	36.09	35.61	35.34
Acid detergent fiber (ADF), % of DM	20.59	19.95	19.91
Crude ash (Ash), % of DM	4.65	4.89	5.11

^1^ The premix provided the following per kg of diets: Cu 0.1–0.5 g, Fe 1–15 g, Zn 1.5–3.0 g, Mn 0.5–3.5 g, Se 5.0–10.0 mg, I 12.0–120.0 mg, Co 5–50 mg, Ca ≥ 200 mg, P ≥ 15 mg, VA 120,000–250,000 IU, VD 10,000–100,000 IU, and VE ≥ 750 IU; abbreviations VA: vitamin A, VD: vitamin D, VE: vitamin E. ^2^ NE_mf_ was determined based on the “China Feeding Standard of Beef Cattle” (NY/T 815-2004 [23]) and dietary gross energy.

**Table 3:** Effects of grape pomace complete pellet feed on growth performance of beef cattle.

Items	Groups			*p*-Value
	G0	G15	G20	
Initial body weight, kg	292.83 ± 8.82	284.17 ± 7.54	285.17 ± 3.44	0.651
Final body weight, kg	342.02 ± 3.61	339.53 ± 4.65	359.11 ± 8.44	0.112
Average daily feed intake, kg/day	9.06 ± 0.11 ^b^	9.40 ± 0.20 ^ab^	9.81 ± 0.13 ^a^	0.036
Average daily gain, kg/day	0.82 ± 0.11 ^b^	0.93 ± 0.07 ^b^	1.23 ± 0.09 ^a^	0.036
Feed conversion, kg/kg	11.50 ± 1.72	10.27 ± 0.79	8.06 ± 0.66	0.099

Within the same row, values marked with distinct lowercase superscripts signify a statistically significant difference (*p* < 0.05). G0: the control group, which consists of the complete pellet feed containing 0% grape pomace; G15: the 15% grape pomace complete pellet feed; G20: the 20% grape pomace complete pellet feed.

**Table 4:** Effects of grape pomace complete pellet feed on serum biochemical indices of beef cattle.

Items	Groups			*p*-Value
	G0	G15	G20	
Urea nitrogen (mmol/L)	1.01 ± 0.11	0.96 ± 0.17	1.75 ± 0.36	0.101
Total Protein (g/L)	61.70 ± 1.18 ^b^	65.60 ± 1.05 ^ab^	70.93 ± 3.14 ^a^	0.049
Albumin (g/L)	34.63 ± 1.17 ^b^	36.27 ± 0.33 ^ab^	38.83 ± 0.83 ^a^	0.034
Globulin (g/L)	27.07±1.96	29.33 ± 0.84	32.1 ± 3.76	0.416
A:G	1.30 ± 0.14	1.24 ± 0.03	1.24 ± 0.15	0.919
A:T	0.56 ± 0.03	0.55 ± 0.01	0.55 ± 0.03	0.899
G:T	0.44 ± 0.03	0.45 ± 0.01	0.45 ± 0.03	0.899
Triglyceride (mmol/L)	0.32 ± 0.06	0.37 ± 0.02	0.32 ± 0.04	0.599
Total cholesterol (mmol/L)	4.54 ± 0.23	5.23 ± 0.42	5.41 ± 0.21	0.183

Abbreviations: A:G, albumin-globulin ratio; A:T, albumin-total protein ratio; G:T, globulin-total protein ratio. Within the same row, values marked with distinct lowercase superscripts signify a statistically significant difference (*p* < 0.05). G0: the control group, which consists of the complete pellet feed containing 0% grape pomace; G15: the 15% grape pomace complete pellet feed; G20: the 20% grape pomace complete pellet feed.

**Table 5:** Effects of grape pomace complete pellet feed on muscle fatty acid composition of beef cattle.

Items (μg/g)	Groups			*p*-Value
	G0	G15	G20	
C8-0	1.04 ± 0.15 ^b^	1.54 ± 0.10 ^a^	1.28 ± 0.05 ^ab^	0.043
C9-0	0.49 ± 0.03	0.66 ± 0.07	0.58 ± 0.01	0.074
C10-0	2.96 ± 0.4	4.25 ± 0.61	4.38 ± 0.27	0.124
C11-0	0.17 ± 0.01	0.20 ± 0.02	0.20 ± 0.01	0.221
C12-0	3.05 ± 0.6	4.59 ± 0.83	5.23 ± 1.29	0.322
C14-0	44.76 ± 7.64	65.49 ± 9.00	71.08 ± 9.35	0.158
C15-0	13.40 ± 3.62	17.19 ± 1.45	18.21 ± 3.77	0.553
C16-0	520.19 ± 13.58 ^b^	595.36 ± 11.37 ^a^	604.92 ± 9.67 ^a^	*p* < 0.01
C17-0	22.79 ± 2.53	27.98 ± 2.33	29.80 ± 2.00	0.162
C18-0	503.91 ± 5.32 ^c^	566.97 ± 5.52 ^b^	608.87 ± 8.53 ^a^	*p* < 0.01
C20-0	4.63 ± 0.46 ^b^	5.99 ± 0.15 ^a^	6.18 ± 0.19 ^a^	0.021
SFA	1117.39 ± 33.11 ^b^	1290.23 ± 24.02 ^a^	1350.72 ± 24.26 ^a^	*p* < 0.01
C14-1	7.15 ± 0.78 ^b^	11.84 ± 1.45 ^ab^	16.10 ± 2.46 ^a^	0.028
C16-1	46.14 ± 2.94	59.63 ± 12.09	68.43 ± 5.36	0.208
C18-1n9c	341.73 ± 8.94 ^b^	366.19 ± 7.24 ^b^	448.52 ± 7.85 ^a^	*p* < 0.01
C20-1(cis-11)	5.52 ± 1.51	4.98 ± 0.83	4.63 ± 0.41	0.832
MUFA	409.88 ± 8.12 ^b^	442.64 ± 21.41 ^b^	537.69 ± 14.65 ^a^	*p* < 0.01
C22-2	5.78 ± 2.32	3.20 ± 0.12	1.91 ± 0.02	0.198
C18-2n6c	528.39 ± 5.69 ^c^	655.71 ± 5.06 ^b^	760.95 ± 3.55 ^a^	*p* < 0.01
C18-3n3	17.69 ± 1.1	22.18 ± 2.59	22.22 ± 2.77	0.339
C18-3n6	2.86 ± 0.29 ^b^	3.75 ± 0.19 ^a^	3.68 ± 0.05 ^a^	0.036
C20-2	11.28 ± 1.76	11.8 ± 0.53	12.89 ± 0.69	0.618
PUFA	566.00 ± 6.77 ^c^	696.64 ± 7.91 ^b^	801.65 ± 2.94 ^a^	*p* < 0.01

SFA: saturated fatty acid, summing the contents of all saturated fatty acids detected in each sample; MUFA: monounsaturated fatty acid, summing the contents of all monounsaturated fatty acids detected in each sample; PUFA: polyunsaturated fatty acid, summing the contents of all polyunsaturated fatty acids detected in each sample. Within the same row, values marked with distinct lowercase superscripts signify a statistically significant difference (*p* < 0.05). G0: the control group, which consists of the complete pellet feed containing 0% grape pomace; G15: the 15% grape pomace complete pellet feed; G20: the 20% grape pomace complete pellet feed.

**Table 6:** Effects of grape pomace complete pellet feed on economic returns.

Items	Groups		
	G0	G15	G20
Feed price, CNY/kg	2.60	2.09	2.04
Total intake, kg/head	492.70	532.17	562.51
Total feed cost, kg/head	1281.02	1112.24	1147.52
Weight gain income, kg/head	1106.62	1245.85	1663.73
Beef cattle prices, CNY/kg	22.50	22.50	22.50
Gross profit, CNY/kg	−174.4	133.61	516.21

## Data Availability

The raw sequence data is accessible under the National Center for Biotechnology Information (NCBI) SRA accession number PRJNA1192575.

## Supplementary Materials

The following supporting information can be downloaded at https://www.mdpi.com/article/10.3390/ani15070930/s1, Table S1: Effects of grape pomace complete pellet feed on rumen fungal alpha diversity index. Table S2: Effects of grape pomace complete pellet feed on rumen fungal flora composition of beef cattle (at the Phylum Level). Table S3: Effects of grape pomace complete pellet feed on rumen fungal flora composition of beef cattle (at the family level). Table S4: Effects of grape pomace complete pellet feed on rumen fungal flora composition of beef cattle (at the genus level).
