# Supplementary material for: Effects of Grape Pomace Complete Pellet Feed on Growth Performance, Fatty Acid Composition, and Rumen Fungal Composition in Beef Cattle
Source: Animals (Basel). 2025 Mar 24;15(7):930. doi: 10.3390/ani15070930 (PMC11988095; doi:10.3390/ani15070930)
Supplement: Supplementary file 1 [file animals-15-00930-s001.zip › animals-3470751-supplementary.pdf]

animals

MDPI

Article

Effects of Grape Pomace Complete Pellet Feed on Growth Performance, Fatty Acid Composition, and Rumen Fungal Composition in Beef Cattle

Meimei Teng[1], Yuanqiu Li[1], Jiangjiao Qi[1], Wenda Wu[1,2,3], Xinchang Sun[1], Chengze Gao[1], Xia Zhang[1], Tursunay Mamtimin[1,4*] and Jiangchun Wan[1,4*]

[1] Xinjiang Key Laboratory of Grassland Resources and Ecology, College of Grassland Science, Urumqi 830052, China; tengmeimei@outlook.com (M.T.); liyuanqiunord@163.com (Y.L.); 15109936697@163.com (J.Q.); wuwenda@hfut.edu.cn (W.W.); sunxinchang2109@outlook.com (X.S.); gao2645024183@outlook.com (C.G.); zhangxia7938@outlook.com (X.Z.); tuexay18@lzu.edu.cn (T.M.); xjau_wjczy@163.com (J.W.)
[2] School of Food and Biological Engineering, Engineering Research Center of Bio-Process, Hefei University of Technology, Hefei 230009, China; wuwenda@hfut.edu.cn (W.M.)
[3] Department of Chemistry, Faculty of Science, University of Hradec Kralove, 500 03 Hradec Kralove, Czech Republic; wuwenda@hfut.edu.cn (W.M.)
[4] Postdoctoral Station of Grassland Science, Urumqi 830052, China; xjau_wjczy@163.com (J.W.); tuexay18@lzu.edu.cn (T.M.)
* Correspondence: tuexay18@lzu.edu.cn (T.M.); xjau_wjczy@163.com (J.W.)

Supplementary Materials:

Table S1. Effects of grape pomace complete pellet feed on rumen fungal alpha diversity index

Items	Groups		
	G0	G15	G20
Chao1	101.53±9.93[a]	72.37±7.73[a]	91.45±12.23[a]
Shannon	3.55±0.18[a]	3.26±0.46[a]	3.70±0.22[a]

Note: Chao1 index and Shannon in α diversity index do not conform to normal distribution, so Kruskal-Wallis is used to test P value.

Within the same row, values marked with distinct lowercase superscripts signify a statistically significant difference ($P<0.05$). G0: the control group, which consists of the complete pellet feed containing 0% grape pomace; G15: the 15% grape pomace complete pellet feed; G20: the 20% grape pomace complete pellet feed.

Table S2 Effects of grape pomace complete pellet feed on rumen fungal flora composition of beef cattle (at the Phylum Level)

Items	Groups		
	G0	G15	G20
Ascomycota	77.09±2.12[b]	81.97±1.00[ab]	83.33±0.74[a]
Mucoromycota	9.19±1.96[b]	14.73±1.09[a]	9.68±0.64[b]
Neocallimastigomycota	4.15±1.55[a]	1.39±0.14[a]	2.70±1.63[a]
Basidiomycota	2.92±0.82[a]	0.79±0.33[a]	1.75±0.62[a]
Others	6.64±1.27[a]	1.11±0.08[b]	2.55±0.25[b]

Within the same row, values marked with distinct lowercase superscripts signify a statistically significant difference ($p < 0.05$). G0: the control group, which consists of the complete pellet feed containing 0% grape pomace; G15: the 15% grape pomace complete pellet feed; G20: the 20% grape pomace complete pellet feed.

Table S3 Effects of grape pomace complete pellet feed on rumen fungal flora composition of beef cattle (at the family level)

Items	Groups		
	G0	G15	G20
Aspergillaceae	73.3±2.68[a]	72.54±2.47[a]	71.38±2.93[a]
Lichtheimiaceae	9.24±1.26[b]	14.31±1.11[a]	10.6±1.08[ab]
Neocallimastigaceae	2.82±0.84[a]	0.73±0.47[a]	2.36±1.78[a]
Saccharomycetaceae	1.53±0.41[a]	3.13±0.25[a]	1.28±0.71[a]
Trichocomaceae	0.93±0.08[a]	1.66±1.60[a]	2.85±2.23[a]
Wallemiaceae	0.97±0.10[a]	0.28±0.14[a]	0.73±0.25[a]
others	11.20±0.96[a]	7.35±0.40[a]	10.80±1.37[a]

Within the same row, values marked with distinct lowercase superscripts signify a statistically significant difference ($p < 0.05$). G0: the control group, which consists of the complete pellet feed containing 0% grape pomace; G15: the 15% grape pomace complete pellet feed; G20: the 20% grape pomace complete pellet feed.

Table S4 Effects of grape pomace complete pellet feed on rumen fungal flora composition of beef cattle (at the genus level)

Items	Groups		
	G0	G15	G20
Penicillium	52.84±1.11[a]	48.48±4.69[a]	41.02±3.84[a]
Aspergillus	17.52±1.48[b]	19.8±3.26[b]	27.28±1.08[a]
Rhizomucor	4.27±0.97[b]	10.22±0.33[a]	8.25±0.5[a]
Lichtheimia	3.96±1.19[a]	3.85±1.08[a]	2.31±0.6[a]
Saccharomyces	1.53±0.41[a]	3.13±0.25[a]	1.28±0.71[a]
Thermomyces	1.33±0.4[a]	2.66±1.14[a]	3.18±2.04[a]
Wallemia	0.97±0.1[a]	0.28±0.14[a]	0.73±0.25[a]
Others	17.57±2.58[a]	11.58±1.99[a]	15.03±1.62[a]

Within the same row, values marked with distinct lowercase superscripts signify a statistically significant difference ($p < 0.05$). G0: the control group, which consists of the complete pellet feed containing 0% grape pomace; G15: the 15% grape pomace complete pellet feed; G20: the 20% grape pomace complete pellet feed.
